# The association between fat-to-muscle ratio and metabolic disorders in type 2 diabetes

**DOI:** 10.1186/s13098-021-00748-y

**Published:** 2021-11-10

**Authors:** Dixing Liu, Jiana Zhong, Yuting Ruan, Zhen Zhang, Jia Sun, Hong Chen

**Affiliations:** Department of Endocrinology, Zhujiang Hospital, Southern Medical University, 253 industrial avenue, Guangzhou, 510282 Guangdong China

**Keywords:** Type 2 diabetes mellitus, Fat-to-muscle ratio, Metabolic disorders, Body composition

## Abstract

**Background:**

Altered body composition is known to be related to abnormal metabolism. The aim of this study was to determine the association between the fat-to-muscle ratio (FMR) and metabolic disorders in type 2 diabetes (T2DM) population.

**Method:**

In total, 361 T2DM participants aged ≥ 18 years were included in our research. A bioelectrical impedance analyzer was applied to measure fat mass and muscle mass. FMR was calculated as body fat mass (kg) divided by muscle mass (kg). The performance of FMR to assess metabolic disorders in T2DM was conducted using ROC curves. The independent association between FMR and metabolic syndrome (MS) was tested by logistic regression analysis.

**Results:**

The FMR was significantly higher in patients with MS than in those without MS (p < 0.001). The optimal FMR cutoff point for identifying MS was higher in females than in males (0.465 vs. 0.296, respectively). In addition, the areas under the ROC curve (AUCs) for the evaluation of MS by FMR, fat mass, muscle mass, BMI and waist circumference were further compared, indicating that the AUC of FMR (0.843) was the largest among the five variables in females, but the AUC of waist circumference (0.837) was still the largest among other variables in males. Based on the derived FMR cutoff point, patients with a high FMR exhibited more cardiometabolic risk indicators (all p < 0.05). Using a low FMR as a reference, the relative risk of a high FMR for MS was 2.861 (95% CI 1.111–7.368, p = 0.029) in males and 9.518 (95% CI 2.615–34.638, p = 0.001) in females following adjustment for confounding factors.

**Conclusions:**

The fat-to-muscle ratio is independently and positively associated with metabolic disorders in T2DM. FMR may serve as an optimal method for screening T2DM patients coupled with a high risk of abnormal metabolism, especially in females, providing a new perspective for the prevention and treatment of cardiovascular complications in Chinese type 2 diabetes.

## Introduction

Metabolic syndrome (MS), a clustering of metabolic abnormalities that includes impaired glucose metabolism, abdominal obesity, dyslipidemia and/or hypertension, is now considered a complex risk factor for type 2 diabetes (T2DM) and cardiovascular disease (CVD) events [1]. Currently, as many as 68.1% of T2DM patients are reported to have MS [2]. In comparison with non-MS patients, those in the T2DM population who coexist with MS were more likely to develop CVD events [3–5] and have an enhanced risk of microvascular complications [6]. In addition, MS can predict the progression of diabetic nephropathy among T2DM population [7]. Thus, early identification of someone at high risk for multiple severe metabolic disorders, especially in T2DM patients, is of paramount importance and represents an effective preventive measure for the development of cardiovascular complications over time in this population.

Obesity, especially abdominal obesity, is generally recognized to be the underlying factor in the development and progression of MS [8]. Although waist circumference and body mass index (BMI) are widely used to evaluate obesity, they have several limitations in evaluating cardiometabolic risks because they cannot quantify body fat and distinguish different body compositions. Recently, with the extensive application of body composition analysis, body fat mass has been considered an effective parameter for assessing the degree of fat accumulation in the body and plays a central role in metabolic disorders [9]. Additionally, excessive adipose tissue was further identified to be accompanied by decreased skeletal muscle quantity [10], known as sarcopenic obesity. This combined phenomenon was more closely related to insulin resistance [11], especially in T2DM patients [12]. Thus, a decline in skeletal muscle mass combined with an increase in body fat may cause a dual metabolic burden, resulting in a higher probability of developing severe metabolic disorders. However, neither body fat nor skeletal muscle mass alone provided all the information required for the assessment of complex body composition changes among the T2DM population. The fat-to-muscle ratio (FMR), integrating two anthropometric indices, might be a proper assessment of the combined effect of body fat accumulation and skeletal muscle mass.

A growing body of studies on chronic metabolic diseases have been carried out on FMR. A high FMR value was reported to be an adverse factor for fatty liver disease [13] and cardiac events [14]. In some studies, FMR was additionally instructed to be associated with MS and insulin resistance among the general population [15, 16]. Park J et al. also indicated that the muscle-to-fat ratio could be a predictor of MS in Korean adults [17]. However, considering the limitations of the study population, much uncertainty still exists concerning the link between FMR and multiple metabolic disorders in T2DM. In addition, the effect of FMR on metabolic disorders in diabetic patients remains absent, and the cutoff point to assess MS based on FMR in T2DM may differ significantly from the general population. Accordingly, our present research intends to explore the correlation between FMR and multiple metabolic disorders, determining the optimal FMR cutoff points for evaluating MS in T2DM.

## Research design and methods

### Study participants

Overall, 361 T2DM patients were selected from Zhujiang Hospital, Southern Medical University from March 2019 to January 2021. The inclusion criteria included age ≥ 18 years and the presence of T2DM according to the 1999 WHO criteria. The exclusion criteria included acute complications of diabetes, e.g., lactic acidosis and diabetic ketoacidosis; a history of malignancy; severe kidney or liver dysfunction; edema, thyroid dysfunction or Cushing syndrome; and pregnancy or lactation. Participants who had bariatric surgery, received weight loss medication (including glucagon-like peptide 1 receptor agonist) or were currently using steroids were also excluded. This research was approved by the ethics committee of Zhujiang Hospital, Southern Medical University.

### Anthropometric and biochemical indicators

Each participant was given a physical examination by a trained physician with measurements of waist circumference, height, and weight. And Body mass index (BMI) was calculated based on weight and height. BMI = weight (kg)/height (m)^2^. Then, blood pressure was measured in duplicate after at least 5 min of rest, and the measurements were averaged. A bioelectrical impedance analyzer (BIA) (Jawon Medical Co., Ltd., Korea) was applied to determine body composition, which is now recognized as a reproducible, convenient and noninvasive method for body composition evaluation [18]. In addition, previous studies have shown that BIA delivers comparable measurements and high accuracy compared with computed tomography (CT) and dual-energy X-ray absorptiometry (DXA) when assessing fat mass and muscle mass [19, 20]. Hence, the following parameters were used: body fat mass, muscle mass, and body fat percentage. The fat-to-muscle ratio (FMR) was calculated as body fat mass (kg) divided by muscle mass (kg).

Samples of venous blood were collected after the 10-h overnight fast. Serum concentrations of fasting glucose, total cholesterol (TC), triglycerides, low-density lipoprotein cholesterol (LDL-C) and high-density lipoprotein cholesterol (HDL-C) were measured using a biochemical autoanalyzer. Glycated hemoglobin (HbA1c) and fasting C-peptide were also detected in the laboratory of Zhujiang Hospital, Southern Medical University. Homeostasis model assessment 2-insulin resistance (HOMA2-IR) was further calculated using HOMA2 Calculator software, V 2.2.3 [21].

### Definition of metabolic syndrome

Metabolic syndrome (MS) was defined according to the *Guidelines for Prevention and Treatment of Dyslipidemia in Chinese Adults* (revised in 2016). The detailed components of MS were defined using the following criteria: (1) abdominal obesity: waist circumference ≥ 90 cm in males or ≥ 85 cm in females; (2) hypertension: blood pressure level of 130/85 or higher or previous diagnosis of hypertension; (3) hyperglycemia: fasting plasma glucose ≥ 6.10 mmol/L, 2-h plasma glucose ≥ 7.8 mmol/L, or diagnosis of T2DM; (4) high triglyceride: the fasting triglyceride level ≥ 1.70 mmol/L; and (5) low HDL-C: the HDL-C level ≤ 1.04 mmol/L. Patients who had any 3 of the 5 above items were diagnosed with MS.

### Statistical analysis

Normally distributed variables are presented as the mean ± SD, whereas skewed values are shown as the median [interquartile range]. T-tests or Mann–Whitney U tests were performed to compare the differences between groups. The Chi-squared test was applied to compare the proportions. The performance of FMR to assess MS in T2DM was conducted using receiver operating characteristic (ROC) curves. We calculated the potential cutoff values of FMR based on the maximized Youden index. The patients were divided according to the FMR cutoff point, and then anthropometric and biochemical variables were compared between groups. The correlation between FMR and cardiometabolic risk factors was detected by Spearman correlation analysis. Then, logistic regression was performed to determine the strong relationship between FMR (by cutoff point) and MS after controlling for confounders, such as age, HbA1c, diabetes duration, BMI and waist circumference. The odds ratio (OR) was calculated with a 95% confidence interval (CI) for the presence of MS. Finally, the areas under the ROC curve (AUCs) for the evaluation of MS by fat mass, muscle mass, FMR, BMI and waist circumference were compared. All statistical analyses were performed utilizing SPSS version 26.0 (IBM Corporation, Armonk, NY, USA). And bar plots were generated in GraphPad Prism V8.0 (San Diego, USA). p values < 0.05 were considered statistical differences.

## Results

A total of 361 T2DM patients aged 56.8 ± 11.1 years were enrolled in the research, of which 59.0% (n = 213) were men and 41.0% (n = 148) were women. And the characteristics of participants by sex are summarized in Table 1. Compared to males, significantly higher levels of fat mass and body fat percentage were observed in females, while the muscle mass was obviously lower (all p < 0.001). The mean FMRs for males and females were 0.331 and 0.530, respectively (p < 0.001). There were also significant differences in lipid profile and blood pressure between sexes (all p < 0.05).

**Table 1:** Characteristics among a sample (mean ± SD or frequency (%))

Variables	Total (n = 361)	Sex		*P* value
		Male (n = 213)	Female (n = 148)	
Age (years)	56.8 ± 11.1	54.6 ± 11.1	59.6 ± 10.4	< 0.001
Diabetes duration (years)	6.0 (1.0–10.0)	5.0 (1.0–10.0)	7.0 (2.0–10.0)	0.051
Height (cm)	164.0 ± 8.2	168.2 ± 6.0	157.4 ± 5.9	< 0.001
Weight (kg)	66.6 ± 12.4	70.1 ± 12.4	61.4 ± 10.5	< 0.001
BMI (kg/m^2^)	24.7 ± 3.7	24.6 ± 3.8	24.8 ± 3.5	0.708
Waist circumference (cm)	92.0 ± 10.1	92.7 ± 10.2	91.0 ± 9.9	0.128
Fat mass (kg)	18.1 ± 6.5	16.6 ± 6.6	20.2 ± 5.8	< 0.001
Muscle mass (kg)	44.7 ± 8.2	49.5 ± 6.4	37.8 ± 4.9	< 0.001
Fat-to-muscle ratio	0.412 ± 0.1	0.331 ± 0.1	0.530 ± 0.1	< 0.001
Body fat percentage (%)	26.8 ± 7.1	23.0 ± 5.8	32.3 ± 5.0	< 0.001
Systolic blood pressure (mmHg)	131.2 ± 19.0	129.2 ± 18.3	134.0 ± 19.7	0.018
Diastolic blood pressure (mmHg)	77.0 ± 12.1	78.1 ± 12.2	75.4 ± 11.8	0.040
Total cholesterol (mmol/L)	5.0 ± 1.4	4.9 ± 1.5	5.2 ± 1.3	0.018
Triglycerides (mmol/L)	1.6 (1.1–2.5)	1.6 (1.1–2.9)	1.7 (1.1–2.3)	0.544
HDL-cholesterol (mmol/L)	1.1 ± 0.3	1.0 ± 0.3	1.2 ± 0.3	< 0.001
LDL-cholesterol (mmol/L)	3.0 ± 1.0	2.9 ± 1.1	3.1 ± 1.0	0.023
TG/HDL ratio	1.6 (0.9–2.8)	1.6 (1.0–3.1)	1.5 (0.9–2.2)	0.025
FPG (mmol/L)	8.6 ± 3.9	8.5 ± 3.9	8.8 ± 3.9	0.539
HbA1c (%)	8.9 ± 2.4	8.9 ± 2.5	8.7 ± 2.4	0.395
HOMA2-IR	2.4 ± 1.5	2.3 ± 1.3	2.6 ± 1.6	0.052

*BMI* body mass index, *LDL*- *cholesterol* low-density lipoprotein cholesterol, *HDL*- *cholesterol* high-density lipoprotein cholesterol, *FPG* fasting plasma glucose, *HOMA2*-*IR* homeostasis model assessment 2-insulin resistance

**Fig. 1:**
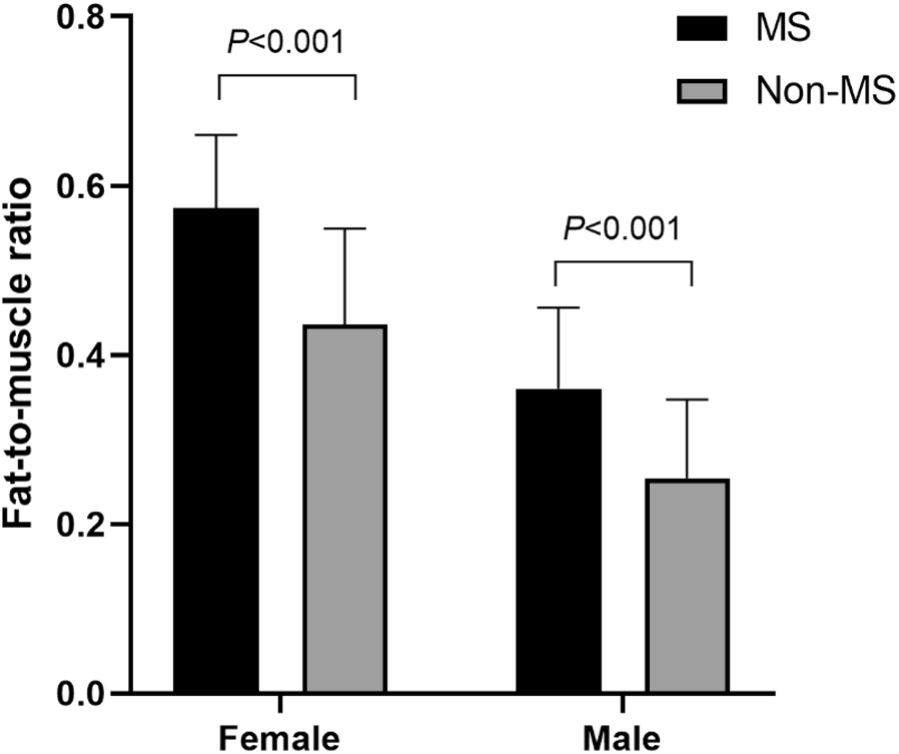
The comparisons of fat-to-muscle ratio in T2DM patients with and without MS

**Fig. 2:**
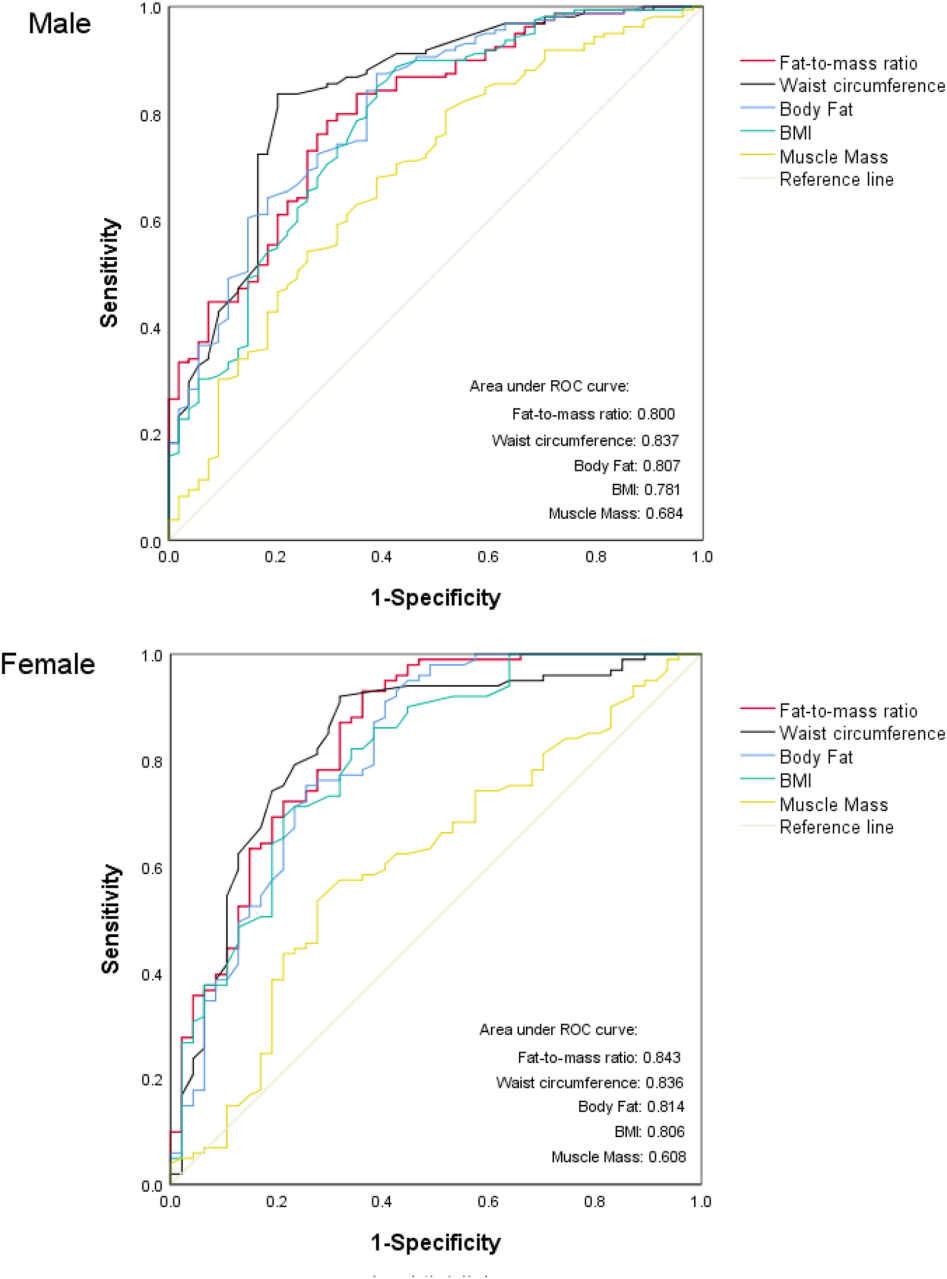
ROC curve of anthropometric for detecting MS in male and female

As shown in Fig. 1, the FMR was significantly higher in subjects with MS than in those without MS (0.360 ± 0.096 vs. 0.245 ± 0.093 in males, p < 0.001; 0.574 ± 0.086 vs. 0.436 ± 0.113 in females, p < 0.001). The ROC curves for the proper performance of FMR in identifying MS in T2DM according to sex are shown in Table 2. The optimal cutoff point of FMR with the largest Youden index was higher in females than in males (0.465 vs. 0.296, respectively). The areas under the ROC curve (AUCs) were 0.843 in women (sensitivity 93.1%, specificity 63.8%) and 0.800 in men (sensitivity 78.6%, specificity 70.4%). Additionally, the AUCs for the evaluations of MS by FMR, fat mass, muscle mass, BMI and waist circumference were further compared, indicating that the AUC of FMR (0.843) was the largest among the five variables in females, while the AUC of waist circumference (0.837) was still the largest among other variables in males (Fig. 2).

**Table 2:** ROC for fat-to-muscle ratio in predicting metabolic syndrome and cut-off points

Parameter	Sex	
	Male	Female
Area under ROC curve (95% CI)	0.800 (0.734–0.867)	0.843 (0.770–0.916)
*P* value	< 0.001	< 0.001
Cut-off point	0.296	0.465
Sensitivity	0.786	0.931
Specificity	0.704	0.638

*ROC* receiver operating characteristic

**Fig. 3:**
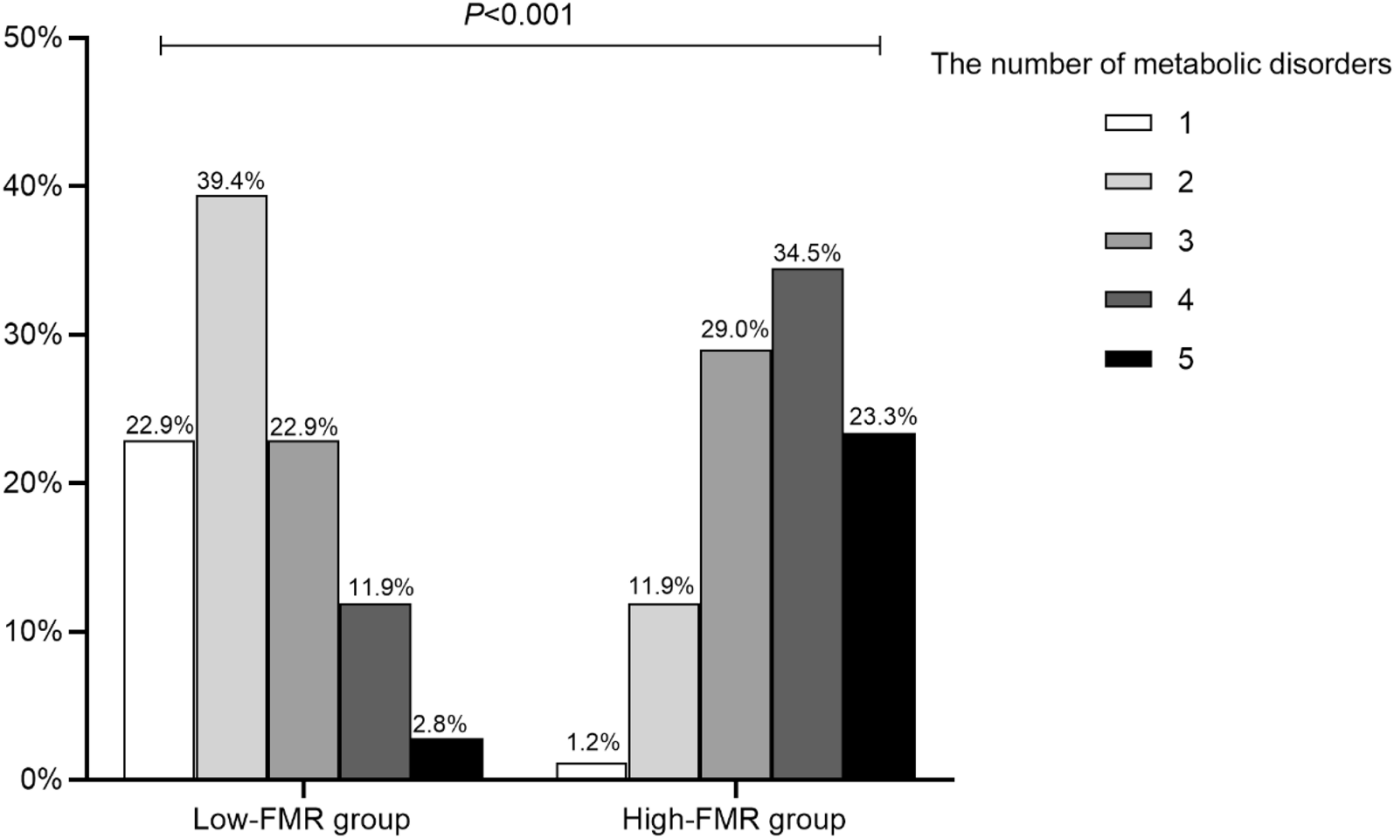
The proportion of metabolic disorders between low-FMR group and high-FMR group. Metabolic disorder was defined as a clustering of metabolic abnormalities that comprise type 2 diabetes, abdominal obesity, hypertension, hypertriglyceridemia, and low HDL-cholesterol. One metabolic disorder was defined as the absence of metabolic abnormalities other than T2DM. Two metabolic disorders were defined as T2DM + any 1 of 4 above items. Three metabolic disorders were T2DM + any 2 of 4 above items. Four metabolic disorders were T2DM + any 3 of 4 above items. Five metabolic disorders contained all the above items. *p* value for the significant difference between groups was determined by χ^2^ test

Based on the derived FMR cutoff points, the patients were divided into a high-FMR group and a low-FMR group. The anthropometric indicators and outcomes for all cardiometabolic risk markers are summarized in Table 3. We found significant differences in anthropometric parameters, blood pressure, triglycerides, HDL-cholesterol, HOMA2-IR and TG/HDL ratio (all p < 0.05), with the exception of muscle mass (p = 0.059), LDL-cholesterol (p = 0.594) and total cholesterol (p = 0.212). Of all enrolled patients, the prevalence of MS was 86.9% in the high-FMR group and only 37.6% in the low-FMR group (p < 0.001). Similarly, the proportion of each MS component was obviously higher in the high-FMR group (all p < 0.001). Patients with high FMR values were more likely to exhibit multiple metabolic disorders (Fig. 3).

**Table 3:** Anthropometric and cardiometabolic risk indicators according to the Sex-specific thresholds of the fat-to-muscle ratio

Variable	Low-FMR group (n = 109)	High-FMR group (n = 252)	*P* value
Weight (kg)	58.5 ± 9.8	70.1 ± 11.6	< 0.001
BMI (kg/m^2^)	21.3 ± 2.5	26.1 ± 3.2	< 0.001
Waist circumference (cm)	84.2 ± 8.3	95.4 ± 8.8	< 0.001
Fat mass (kg)	11.6 ± 3.8	20.9 ± 5.4	< 0.001
Muscle mass (kg)	43.4 ± 8.2	45.2 ± 8.2	0.059
Body fat percentage (%)	19.9 ± 6.1	29.8 ± 5.2	< 0.001
Systolic blood pressure (mmHg)	127.1 ± 17.0	132.9 ± 19.6	0.005
Diastolic blood pressure (mmHg)	75.0 ± 10.7	77.9 ± 12.5	0.039
Total cholesterol (mmol/L)	4.9 ± 1.3	5.1 ± 1.5	0.212
Triglycerides (mmol/L)	1.2 (0.9–1.8)	1.9 (1.3–2.9)	< 0.001
HDL-cholesterol (mmol/L)	1.2 ± 0.3	1.0 ± 0.3	< 0.001
LDL-cholesterol (mmol/L)	2.9 ± 1.0	3.0 ± 1.0	0.594
TG/HDL ratio	1.0 (0.7–1.7)	1.8 (1.1–3.1)	< 0.001
HOMA2-IR	1.8 ± 1.1	2.6 ± 1.5	< 0.001
Hypertension (n, %)	43 (39.4%)	176 (69.8%)	< 0.001
Fatty liver disease (n, %)	39 (36.8%)	179 (74.6%)	< 0.001
Abdominal obesity (n, %)	37 (33.9%)	210 (83.3%)	< 0.001
Hypertriglyceridemia (n, %)	31 (28.4%)	145 (57.5%)	< 0.001
Low HDL-cholesterol (n, %)	33 (30.3%)	142 (56.3%)	< 0.001
Metabolic syndrome (n, %)	41 (37.6%)	219 (86.9%)	< 0.001

The thresholds of fat-to-muscle ratio were derived from ROC analysis, where FMR > 0.296 in male or > 0.465 in female was classified as high-FMR

*FMR* fat-to-muscle ratio

Furthermore, Spearman correlation analysis revealed that FMR was positively associated with waist circumference, BMI, triglycerides, TG/HDL ratio and HOMA2-IR but negatively correlated with HDL-cholesterol in both males and females (all p < 0.05). However, the relationship between FMR and systolic blood pressure was observed only in women (r = 0.242, p = 0.003). In addition, a weak correlation between FMR and HbA1c and diabetes duration was detected in men but not in women (Table 4).

**Table 4:** Spearman correlation analysis of fat-to-mass ratio and other variables

Variables	Male		Female	
	r	*P* value	r	*P* value
Age	− 0.061	0.379	0.244	0.003
Diabetes duration	− 0.151	0.027	0.059	0.476
HbA1c	0.140	0.043	0.056	0.503
BMI	0.778	< 0.001	0.860	< 0.001
Waist circumference	0.699	< 0.001	0.706	< 0.001
Systolic blood pressure	0.080	0.245	0.242	0.003
Diastolic blood pressure	0.107	0.119	0.094	0.258
Total cholesterol	0.098	0.156	0.123	0.137
Triglycerides	0.301	< 0.001	0.361	< 0.001
HDL-cholesterol	− 0.185	0.007	− 0.263	0.001
LDL-cholesterol	0.040	0.565	0.058	0.482
TG/HDL ratio	0.311	< 0.001	0.357	< 0.001
HOMA2-IR	0.372	< 0.001	0.351	< 0.001

Finally, logistic regression analysis was further performed to determine the independent relationship between FMR (by cutoff level) and MS in T2DM patients. In this analysis, using the low-FMR group as the reference, the results showed that the relative risk for MS was 8.249 (95% CI 4.092–16.627, p < 0.001) in men and 22.726 (95% CI 8.507–60.716, p < 0.001) in women with high FMR values following adjustment for age (Model 1). After further adjustments for HbA1c, diabetes duration, waist circumference and BMI, the association between FMR and MS still reached statistical significance, and the relative risks for MS were 2.861 (95% CI 1.111–7.368, p = 0.029) in men and 9.518 (95% CI 2.615–34.638, p = 0.001) in women (Model 2) (Table 5).

**Table 5:** Associations between fat-to-mass ratio (by cut-off level) and metabolic syndrome by logistic regression analysis in different gender

	Male		Female	
	OR (95% CI)	P	OR (95% CI)	P
Model 1	8.249 (4.092–16.627)	< 0.001	22.726 (8.507–60.716)	< 0.001
Model 2	2.861 (1.111–7.368)	0.029	9.518 (2.615–34.638)	0.001

Data are presented as odds ratios (95% confidence interval)

Model 1: adjustment for age

Model 2: Model 1 + adjustment for HbA1c, diabetes duration, BMI and waist circumference

## Discussion

In the current study, the presence of MS in the T2DM population was found to have a higher FMR value than the non-MS population. FMR was independently and positively associated with MS after adjusting for potential confounders. In men, the risk of MS was 2.9-fold higher in the high-FMR group than in the low-FMR group; in women, it was 9.5-fold higher. Additionally, the cutoff points of FMR in identifying MS were higher in females than in males. Finally, the FMR may serve as an optimal parameter for screening individuals at high risk for multiple metabolic disorders in T2DM patients, especially among females.

In recent years, increased body fat mass has been identified to be accompanied by decreased muscle mass, presenting a dual metabolic burden that might emerge as a key driver of metabolic disorders. FMR was established as a robust indicator that can reflect the alteration of body composition caused by body fat and skeletal muscle to some extent. And the possible adverse effects of these alteration on metabolism may be further identified by FMR. A population-based study revealed that the cutoff values of FMR for identifying MS were 0.336 in men and 0.555 in women [15]. However, some discrepancies in body composition between diabetic patients and nondiabetic patients were observed by Chen Y [22, 23] and Pechmann LM et al. [24]. In this study, the cutoff points of FMR were significantly lower in T2DM patients (0.296 in men and 0.465 in women) than in the general population. Our findings indicated that diabetes patients exhibited a lower threshold of FMR for multiple metabolic abnormalities, suggesting the possible effect of T2DM, per se, on body composition, especially exacerbating changes in the FMR. Recently, Wang et al. confirmed that T2DM patients showed lower muscle mass to visceral fat area ratio than subjects without T2DM [25]. Previous reports demonstrated that long-term suboptimal glycemic control was related to protein catabolism in skeletal muscle. The decrease in muscle mass due to enhanced catabolism has been identified to be combined with obesity, thereby changing FMR [26, 27]. However, in the present study, the correlation between FMR and HbA1c was weak in males but not in females. We considered that the HbA1c level reflects the average blood glucose levels only during the past 8–12 weeks and fails to represent the long-lasting glycemic control of T2DM patients. Therefore, HbA1c should be measured multiple times over the long term to further verify the relationship between FMR and glycemic control.

In our study, FMR exhibited positive correlations with blood pressure and serum lipid profile. Higher proportions of hypertension and dyslipidemia were also found among patients with higher FMR levels. Coinciding with our results, a cross-sectional study of 6832 adults in Korea indicated that abdominal obesity coupled with a decline in skeletal muscle mass was associated with hypertension [28]. Similarly, decreased muscle mass and increased fat mass were also demonstrated to be associated with hyperlipidemia [29]. In addition, we also found a positive correlation between FMR and age, which indicated that older patients with T2DM may be more susceptible to abnormal body composition arising from age-related loss of skeletal muscle quantity and accumulation of body fat. This condition is probably because of a decrease in physical activity and altered dietary intake in older adults. However, the unfavorable effects of age may appear to mediate the effects of FMR, especially in the female population, where increased age or even diabetes duration was observed in our research. Thus, we further examined the independent effect of FMR with respect to MS in males and females after adjusting for plausible confounders, including age and diabetes duration. Finally, the statistical correlation between FMR and MS remained, suggesting that the relationship was non-sex-specific and independent of age and diabetes duration. A high FMR level is a risk factor related to metabolic disorders in T2DM.

Although the precise mechanism of the relationship between FMR and metabolic disorders is not clear, previous studies have shown that FMR is closely related to insulin resistance [15]. Significant associations between FMR and HOMA2-IR and the TG/HDL ratio were also detected in the current research. In addition, a recent study indicated that the reduction in muscle quantity and the obesity-related increase in fat mass were correlated with inflammatory cytokines, suggesting that a high FMR level may reflect a state of inflammation [30]. Thus, these factors may contribute to demonstrating the independent association between FMR and metabolic disorders in T2DM. Notably, patients who used statin were also included in the study. Although the proportion of statin use was only 22.7% in all study participants and no statistically significant differences were observed between the low-FMR and high-FMR groups (18.3% in low-FMR group and 24.6% in high-FMR group, p = 0.193, data not shown), this group of drugs is known to have potential adverse effects on muscle. However, statin-induced myopathy was rarely related to sarcopenia, and the effects of statin treatment on muscle parameters remain debatable. Lindstrom I et al., indicated that statin use does not reduce muscle mass or predispose patients to increased sarcopenia [31]. And a prospective study also did not observe an effect of statin therapy on muscle strength during a 6-month follow-up [32]. Conversely, Scott et al. showed a correlation between statin treatment and decreased muscle strength after a 3-year follow-up. But there was no difference in muscle mass [33]. Taken together, there is still no compelling evidence that statin treatment contributes to decreased muscle mass. However, the possible bias caused by statin therapy may be associated with FMR in this study.

Several limitations still exist in this study. As a predictor of multiple metabolic disorders that develop in patients with T2DM, it might be necessary to conduct a longitudinal study in the future. Second, even though the most precise method to assess body composition is MRI or CT, limitations exist on account of their high cost and harmful radiation, which make them difficult to popularize in clinical practice. In contrast, bioelectrical impedance analysis (BIA) developed as a highly accurate method that is suitable for evaluating the body composition. Third, low muscle strength has also been identified as a risk factor for MS in recent studies [34, 35]. However, we did not measure muscle function or strength in this study; therefore, the role of muscle strength in metabolic disorders is perhaps neglected. Thus, combining muscle strength and FMR may improve the assessment of metabolic disorders over FMR alone in the future. Finally, there were no data on inflammatory factors, physical activity, or dietary intake in the present research. More studies are warranted to further discuss the association between these factors and FMR in T2DM patients.

## Conclusions

The fat-to-muscle ratio is independently and positively associated with metabolic disorders in T2DM. The cutoff points of FMR in identifying metabolic disturbances were higher in females than in males. The FMR may serve as an optimal indicator for screening T2DM patients coupled with a high risk of multiple metabolic disorders, especially in the female population, providing a new perspective for the prevention and treatment of cardiovascular complications in Chinese T2DM patients.

## Data Availability

The data used and analyzed throughout this research can be obtained from the corresponding authors.
